# Atomic Force Microscopy Study of the Long-Term Effect of the Glycerol Flow, Stopped in a Coiled Heat Exchanger, on Horseradish Peroxidase

**DOI:** 10.3390/mi15040499

**Published:** 2024-04-04

**Authors:** Yuri D. Ivanov, Ivan D. Shumov, Andrey F. Kozlov, Anastasia A. Valueva, Maria O. Ershova, Irina A. Ivanova, Alexander N. Ableev, Vadim Y. Tatur, Andrei A. Lukyanitsa, Nina D. Ivanova, Vadim S. Ziborov

**Affiliations:** 1 Institute of Biomedical Chemistry, Pogodinskaya Str., 10 Build. 8, 119121 Moscow, Russia; shum230988@mail.ru (I.D.S.); afkozlow@mail.ru (A.F.K.); varuevavarueva@gmail.com (A.A.V.); motya00121997@mail.ru (M.O.E.); i.a.ivanova@bk.ru (I.A.I.); ableev@mail.ru (A.N.A.); ziborov.vs@yandex.ru (V.S.Z.); 2 Joint Institute for High Temperatures of the Russian Academy of Sciences, 125412 Moscow, Russia; 3 Foundation of Perspective Technologies and Novations, 115682 Moscow, Russia; v_tatur@mail.ru (V.Y.T.); andrei_luk@mail.ru (A.A.L.); ninaivan1972@gmail.com (N.D.I.); 4 Faculty of Computational Mathematics and Cybernetics, Moscow State University, 119991 Moscow, Russia; 5 Moscow State Academy of Veterinary Medicine and Biotechnology Named after Skryabin, 109472 Moscow, Russia

**Keywords:** horseradish peroxidase, glycerol, atomic force microscopy, enzyme aggregation

## Abstract

Glycerol is employed as a functional component of heat-transfer fluids, which are of use in both bioreactors and various biosensor devices. At the same time, flowing glycerol was reported to cause considerable triboelectric effects. Herein, by using atomic force microscopy (AFM), we have revealed the long-term effect of glycerol flow, stopped in a ground-shielded coiled heat exchanger, on horseradish peroxidase (HRP) adsorption on mica. Namely, the solution of HRP was incubated in the vicinity of the side of the cylindrical coil with stopped glycerol flow, and then HRP was adsorbed from this solution onto a mica substrate. This incubation has been found to markedly increase the content of aggregated enzyme on mica—as compared with the control enzyme sample. We explain the phenomenon observed by the influence of triboelectrically induced electromagnetic fields of non-trivial topology. The results reported should be further considered in the development of flow-based heat exchangers of biosensors and bioreactors intended for operation with enzymes.

## 1. Introduction

Enzymes have found numerous applications in biotechnology [1] and biomedical science [2]. In nature, enzymes catalyze reactions in living cells [3] and can be employed as catalysts in a wide range of commercially important processes [1]. The list of biotechnological applications of enzymes includes, for instance, food processing, synthesis of pharmaceuticals, paper fabrication, etc. [1,4]. As regards biomedicine, applications of enzymes in biosensors [2] and in diagnostic test kits [5,6] should be mentioned.

Enzyme-based catalysis requires the proper selection and careful maintenance of optimal process conditions since enzymes quickly lose their functional activity at extreme temperatures [7], pH values, ion concentrations, and pressures [8]. This is why biosensors and bioreactors intended for operation with enzymes are often equipped with thermal stabilization systems [9,10]. In these thermal stabilization systems, cylindrically wound pipes (or simply coils) with circulating heat-transfer fluid are often employed [11,12,13]. The use of glycerol as a component of heat-transfer fluids was shown to be promising [14,15]. At that, it should be emphasized that the flow of glycerol in a pipe induces electromagnetic fields due to the so-called triboelectric effect [16,17]. Electromagnetic fields generated upon the flow of glycerol can be quite strong [16], thus representing an important factor influencing the activity of enzymes [18]. The effect of a triboelectrically induced field on an enzyme can take place even after stopping the flow of heat transfer fluid [18].

One of the possible effects of electromagnetic fields on an enzyme is exhibited in the form of a change in its aggregation state upon adsorption onto a solid substrate surface [18,19,20]. This is an important point since surface-adsorbed enzymes are widely employed in biotechnology [1]. Aggregation of proteins, including enzyme proteins, is generally attributed to misfolding or partial unfolding of their polypeptide chains [21,22]. With regard to enzymes, their aggregation can also be related to a change in their hydration [18,19,23,24,25]. In general, aggregation is considered to cause a decrease in the functional activity of enzymes [26]. Colombie et al. demonstrated that inactivation of lysozyme in a bioreactor is accompanied by its aggregation [27]. On the other hand, Gentile et al. emphasized that aggregation of an enzyme can occur in the course of its functioning and does not inevitably imply activity loss [28]. Accordingly, enzyme aggregation and external factors influencing this process require further thorough investigation. In this respect, ultrasensitive methods such as atomic force microscopy (AFM) are of use [18,19,29,30,31] since they allow researchers to reveal even subtle effects of external impacts on enzyme aggregation [31].

Horseradish peroxidase (HRP) is a ~44 kDa enzyme glycoprotein [32,33]. It is widely employed as a component of enzyme-linked immunosorbent assay (ELISA) kits [34] and as a reporter enzyme in biosensors [35]. Furthermore, HRP has found many industrial applications in food technology [36], wastewater purification [37], and biofuel cell fabrication [38,39,40]. At that, the aggregation state of HRP was shown to be influenced by external magnetic [29,30] and electromagnetic fields [18,19,20]. Electromagnetic fields are ubiquitous in industry [41]. As mentioned above, the aggregation state of enzymes can influence their functional activity. This explains the importance of further in-depth investigation of the influence of electromagnetic fields on the aggregation state of HRP.

Our present study reveals a considerable 40 min after-effect (the so-called long-term effect [19]) of the glycerol flow in a cylindrically coiled heat exchanger on the aggregation state of HRP after incubation of its solution near the outer side of the coiled section, which has been covered with a grounded shield. In other words, the glycerol flow has been stopped prior to the incubation of the enzyme. The enzymatic activity of HRP has been found unaffected. Nevertheless, considerably increased content of aggregated enzyme has been revealed by AFM on the surface of mica substrates after the incubation of the enzyme in our experimental setup. Since the coil in the setup has been ground-shielded, the phenomenon observed can be explained by the occurrence of the so-called knotted electromagnetic fields [20].

## 2. Materials and Methods

### 2.1. Chemicals and Enzyme

Both the HRP enzyme (peroxidase from horseradish; cat. #6782) and its substrate 2,2′-azino-bis(3-ethylbenzothiazoline-6-sulfonate) (ABTS; cat. #A-1188) were purchased from Sigma (St. Louis, MO, USA). In AFM experiments, we used 2 mM Dulbecco’s modified phosphate buffered saline (PBSD buffer) was prepared by dissolving the salt mixture purchased from Pierce (USA) in the appropriate amount of ultrapure water. In spectrophotometry experiments, we used buffer salts and hydrogen peroxide purchased from Reakhim (Moscow, Russia). In all experiments, deionized ultrapure water (with 18.2 MΩ × cm resistivity), obtained with a Simplicity UV system (Millipore, Molsheim, France), was used.

The enzyme samples tested in the experiments represented 0.1 µM HRP solutions in 2 mM PBSD.

### 2.2. Experimental Setup and Enzyme Treatment

**Figure 1:**
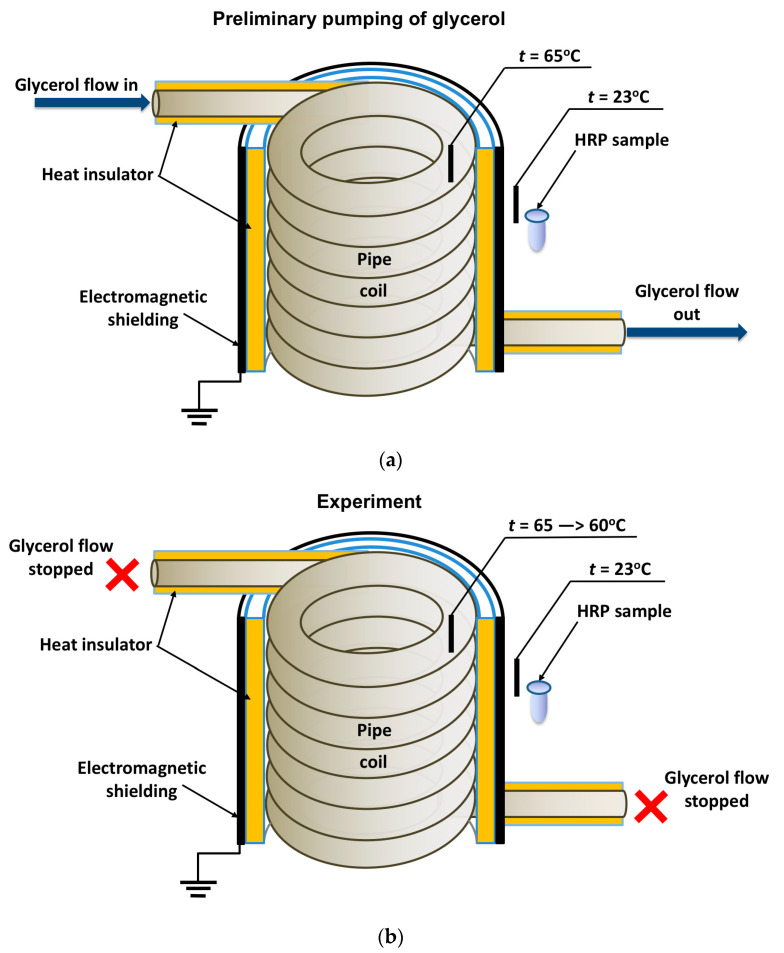
Schematic of the experimental setup illustrating the preliminary pumping of glycerol (**a**) and the incubation of the enzyme solution (working sample) near the coiled section of the heat exchanger after stopping the glycerol flow (**b**). Black rectangles indicate thermocouple-based thermometers used for temperature monitoring.

In general, the experimental setup was similar to that used in our previous study [19], but the enzyme was incubated near the heat exchanger’s coiled section. The location of the tested HRP solution (working sample) is schematically shown in Figure 1.

The cylindrically wound polymeric pipe modeled the heat exchanger. Prior to the experiment, warm (65 °C) glycerol (Glaconchemie GmbH, Merseburg, Germany) had been continuously pumped through this pipe at a flow rate of 9 L/s for 40 min. The glycerol temperature was monitored using an FY-10 digital thermometer, whose sensor was fixed on the inner side of the coil (Figure 1a,b). The use of warm glycerol provided the necessary fluidity of the glycerol and, hence, the desired flow rate [18,19].

After the 40-min-long pumping, the glycerol flow was stopped, and a working sample of the enzyme solution (1 mL in a 1.7 mL polypropylene single-use Eppendorf-type test tube) was incubated near (at a 2 cm distance) the heat-insulating cover of the ground-shielded heat exchanger for 40 min. The heat insulating cover allowed us to avoid undesired heating of the enzyme sample (which can affect its activity [36,42]) so that the enzyme incubation was performed at room temperature (23 °C). At that, the temperature of glycerol within the coil decreased from 65 to 60 °C during the incubation of the sample. The temperature of both the coil and the sample was controlled with FY-10 digital temperature sensors. A control sample of the enzyme (1 mL in a 1.7 mL polypropylene single-use Eppendorf-type test tube) was placed three meters away from the experimental setup. Both the working and the control enzyme samples were then subjected to AFM and spectrophotometry analysis.

### 2.3. Preparation of Substrates and Atomic Force Microscopy Measurements

Enzymes from both the working and the control samples were directly adsorbed onto 7 mm × 15 mm pieces of freshly cleaved mica. These mica pieces were used as AFM substrates, according to Kiselyova et al. [43], as described previously [18,19,20]. Each substrate was immersed into the 1.7 mL Eppendorf-type tube. The test tube contained 0.8 mL of the respective enzyme sample. The substrates were incubated in the test tubes for ten minutes at room temperature and 600 rpm. After the incubation, the substrates were washed with deionized water and dried in air. Each of the so-prepared substrates was then scanned with a Titanium atomic force microscope (NT-MDT, Zelenograd, Russia; the microscope pertains to the equipment of the “Human Proteome” Core Facility of the Institute of Biomedical Chemistry, supported by the Ministry of Education and Science of the Russian Federation, Agreement 14.621.21.0017, unique project ID: RFMEFI62117X0017). The microscope was equipped with NSG10 cantilevers (TipsNano, Zelenograd, Russia; resonant frequency 47–150 kHz, force constant from 0.35 to 6.1 N/m). The AFM scanning was carried out in a semi-contact mode in air at a temperature of 25 °C. The size of each scan was either 1 × 1 µm^2^ or 2 × 2 µm^2^, and the scanning resolution was 256 × 256. For each substrate, at least 16 scans in different areas of the substrate were obtained.

For each AFM substrate, we calculated the total number of objects visualized in the AFM images, which was ≥200. The distributions of the relative number of objects with height *ρ*(*h*) (density functions) were calculated for each sample studied as described previously by Pleshakova et al. [44]:

*ρ*(*h*) = (*N_h_*/*N*) × 100%, (1)

where *N_h_* is the number of objects with height *h*, and *N* is the total number of the visualized objects. In blank experiments, which were performed with protein-free buffer instead of enzyme solution in order to estimate the number of non-specifically adsorbed objects on the substrate surface, no objects with heights exceeding 0.5 nm were registered.

For each sample studied, the absolute number of AFM-visualized particles, normalized per 400 µm^2^, was also calculated according to Pleshakova et al. [44]:

*N*_400_ = 400 *× N/A*, (2)

where *A* is the summarized area of all scans obtained.

The microscope operation and further processing of AFM images (alignment, flattening correction, export to ASCII format, etc.) were performed with a standard NOVA Px 3.5.0 rev. 20364 software (NT-MDT, Moscow, Zelenograd, Russia). The number of visualized particles was determined using custom software, which was developed in IBMC [18].

### 2.4. Spectrophotometry Analysis

We carried out spectrophotometric measurements in order to assess whether the enzymatic activity of HRP against ABTS was affected in our experiment. We employed the technique developed by Sanders et al. [45]. The spectrophotometric measurements were performed in phosphate–citrate buffer [45] at an acidic pH of 5.0, as was recommended by Drozd et al. [46]. Indeed, introducing citrate [46] or acetate [47] ions was reported to promote ABTS oxidation [46], while the pH is usually maintained between 4.2 [47] and 5.0 [45,46]. The measurements were performed at 405 nm in a 1 cm quartz spectrophotometric cell for 5 min. The solution in the cell contained 1 nM HRP, 0.3 mM ABTS, and 2.5 mM hydrogen peroxide [45].

## 3. Results

**Figure 2:**
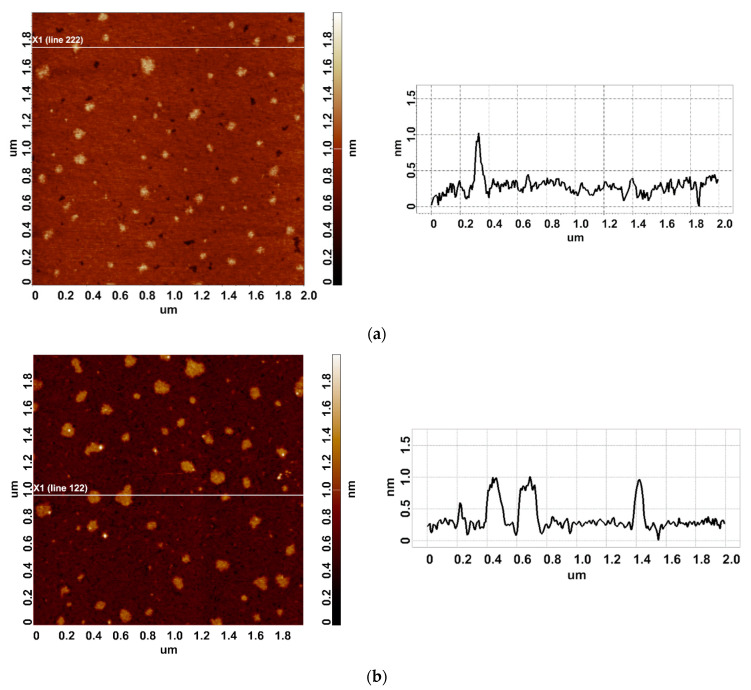
Typical AFM images of HRP particles adsorbed on mica from the control (**a**) and the working (**b**) enzyme samples. The right panels display the cross-section profiles corresponding to the lines in the AFM images shown in the respective left panels.

In our experiments, the working enzyme sample was incubated near the coiled heat exchanger with a stopped-flow of glycerol—namely, at a 2 cm distance from the outer side of the coil. In this location, the temperature was 23 °C. At that, within the coil, the temperature decreased from 65 °C to 60 °C during the experiment (Figure 1b). Accordingly, the heat insulation allowed us to avoid temperature effects on the enzyme [42]. At the same time, the control enzyme sample was kept three meters away from the experimental setup. Figure 2 displays typical AFM images of HRP particles adsorbed onto mica substrates after the incubation in either the working or the control enzyme sample.

**Figure 3:**
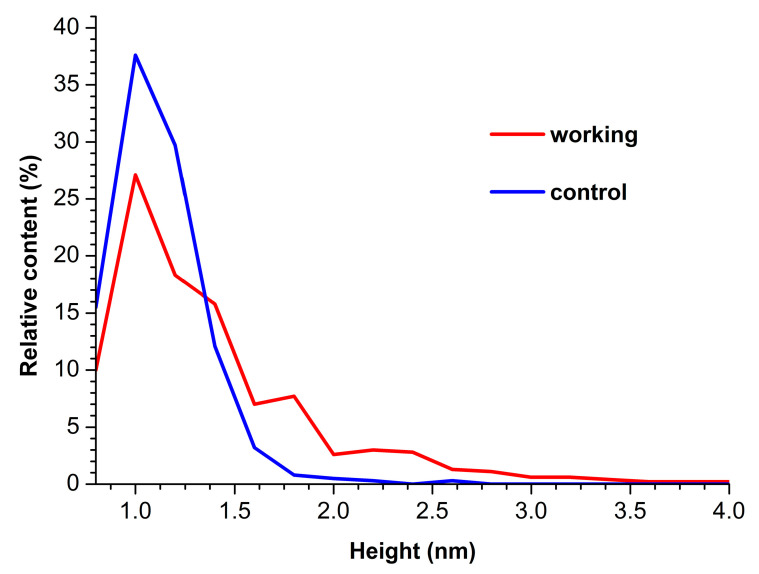
Density function plots obtained for control (blue curve) and working (red curve) HRP samples.

In both AFM images presented, compact objects with heights from 1 to 1.2 nm can be distinguished. These values correspond to the height of mica-adsorbed HRP [20]. In blank experiments, no objects of >0.5 nm height were visualized. In order to find out the difference in enzyme adsorption between the working sample and the control one, the density function (*ρ*(*h*)) plots obtained for these samples have been analyzed. These *ρ*(*h*) plots are shown in Figure 3.

The curves shown in Figure 3 indicate that for the control enzyme sample, the maximum of the respective density function corresponds to *h_max_*(*control*) = 1.0 ± 0.2 nm, while its width at half-height (WHH [44]) makes up *WHH*(*control*) = 0.5 nm. As was mentioned above, the height of AFM images of monomeric HRP is 1.0 to 1.2 nm. Accordingly, objects of this height observed on the mica surface can be attributed to HRP monomers.

For the working enzyme sample, the maximum of the respective density function was similar to that observed for the control one: *h_max_*(*working*) = 1.0 ± 0.2 nm. At that, the distribution obtained for the working sample was considerably broader: *WHH*(*working*) = 0.7 nm. The broader *ρ*(*h*) distribution obtained for the working sample indicates an increased aggregation of HRP in it—as compared with the control sample. The higher objects should be attributed to aggregated enzymes [20]. Furthermore, an increased contribution of particles with heights > 1.4 nm was observed in comparison to the control sample, and this is the second fact indicating increased aggregation of HRP.

**Figure 4:**
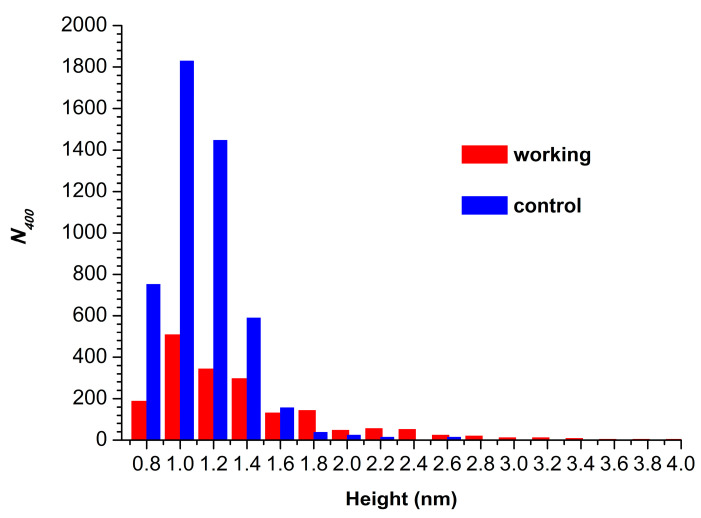
Histograms of *N*_400_ plotted vs. height for control (blue bars) and working (red bars) HRP samples.

Figure 4 displays histograms of absolute number of AFM-visualized particles, normalized per 400 µm^2^ (*N*_400_), plotted vs. height.

The *N*_400_ value obtained for the working sample was *N*_400_(*working*) = 1876 particles/400 µm^2^. For the control sample, the *N*_400_ value was higher (though of the same order of magnitude), amounting to 4867 particles/400 µm^2^.

As regards the spectrophotometry results, no difference in the activity of the enzyme against ABTS in the working and the control samples was observed.

## 4. Discussion

In the Introduction, we have mentioned the long-term effect, which was observed after the incubation of the HRP enzyme near the inlet and the outlet linear sections of the heat exchanger with stopped flow of glycerol [19]. In the previously studied case, both the adsorption properties and the enzymatic activity of HRP were found to be affected. Now, we present the results of our recent experiments performed in the continuation of our studies in this respect. In the experiments reported herein, we have found that the incubation of HRP in the vicinity of the outer side of the ground-shielded cylindrically coiled section of the heat exchanger with stopped glycerol flow also leads to a long-term effect on the enzyme aggregation state. At that, we note once again that no heating of the working enzyme sample has been observed during the experiment. This was achieved owing to proper thermal shielding of the coil with warm glycerol. Inside the coil, the temperature decreased from 65 °C to 60 °C during the 40 min sample incubation. Avoiding the heating of the enzyme sample is a crucial point since the enzymatic activity of HRP is known to increase at elevated (45 °C to 50 °C) temperatures [42]. At the same time, no effect on the enzymatic activity of HRP against its substrate ABTS has been revealed.

As regards the AFM study of the aggregation of the enzyme upon its adsorption onto mica substrate, the incubation of the enzyme sample in the vicinity of the heat exchanger with stopped glycerol flow has been found to cause an increase in the relative content of aggregated HRP in comparison with the control enzyme sample (Figure 3). Below, we discuss the factors which can contribute to this process.

We must emphasize that in enzyme adsorption studies, enzyme-enzyme, enzyme-substrate, and enzyme-solvent interactions should be taken into consideration [48]. The combination of these interactions will determine the aggregation state of the adsorbed enzyme in each particular case.

The first factor to be considered is the electrostatic interaction between the enzyme globule and the substrate surface. Adsorption of enzymes is often considered as electrostatically-driven process [49]. Our adsorption experiments were performed at pH 7.4 when the bare mica surface bears a strong negative charge [50]. The most abundant HRP isoform was reported to have an isoelectric point at pH close to 9 [33], thus bearing a positive charge under our experimental conditions. Accordingly, electrostatic interactions are expected to have a considerable contribution to the adsorption of HRP onto mica in our case. Nevertheless, other types of interactions should also be taken into consideration, particularly in order to explain the difference in the adsorption behavior of the enzyme in the working and the control samples.

With respect to the aggregation of an enzyme, both electrostatic and van der Waals forces should be taken into consideration [51,52]. Van der Waals interactions are particularly important in the case of large enzyme particles [53,54]. This is quite important in the case of HRP, which is prone to aggregation at high (micromolar) concentrations [20,55]. In fact, this is the reason we have performed the adsorption experiments at a lower (0.1 µM) HRP concentration [20]. Namely, under our experimental conditions, untreated HRP is predominantly adsorbed onto mica in its monomeric form [20]. Here, we must note that, despite the relatively low concentration of the enzyme in the bulk solution, its concentration in the near-surface layer increases throughout the binding with the substrate surface [56], thus enhancing the importance of van der Waals interactions. The latter represents the second factor influencing the adsorption and aggregation of HRP.

The third important factor is hydration repulsion, which was also reported to contribute to protein adsorption [57]. In aqueous enzyme solutions, the hydration shells formed around enzyme globules should be considered with respect to enzyme-solvent interactions. Duinhoven et al. emphasized the role of hydration layers on both the substrate surface and the outer surface of the enzyme globule with regard to exposure to hydrophobic interaction sites of the enzyme [58]. These authors noted that dehydration of hydrophobic interfaces promotes enzyme adsorption. Indeed, hydrophobic interactions typically lead to strong adsorption [59], often playing the determining role in this process [58]. Moreover, hydrophobic enzyme-enzyme interactions were reported to be the cause of formation of aggregates [21]. Accordingly, the re-arrangement of outer hydration shells of individual enzyme globules can be the very factor favoring their adsorption and aggregation.

According to the above-discussed considerations, the increase in the degree of HRP aggregation, which was observed in working experiments, can be well explained by changes in the hydration shells of the enzyme globules [60]. This change can also occur in the course of the 40-min-long incubation of the enzyme sample near the heat exchanger with stopped glycerol flow [19]. The change in the outer hydration shells of HRP enzyme globules can influence their interactions with each other [61], as well as with the surface of the mica substrate [48,62]. This effect could be associated with long-lived perturbations resulting from the formation of nanobubble clusters [63]. Bunkin et al. [63] observed this phenomenon using a fluorescence-based technique and explained it by an electromagnetic perturbation induced by an external electromagnetic field in aqueous solutions. In our work, electromagnetic radiation can occur at the expense of the triboelectric generation of charge, as was noted in the Introduction [16,17].

Of note, Beaufils et al. [62] have mentioned electromagnetic radiation within the list of factors influencing protein structure. Accordingly, adsorption and aggregation properties of the HRP glycoprotein can also be affected in this way. It should be emphasized that the coiled heat exchanger has been ground-shielded. Nevertheless, the long-term effect on the enzyme has been clearly observed (see Figure 3). This phenomenon can be explained by the influence of electromagnetic fields of non-trivial topology, which are known as “knotted” electromagnetic fields [20].

The effect of external electromagnetic fields on enzymes varies depending on the enzyme type, structure, and composition [64,65]. HRP studied herein is a heme-containing enzyme [33]. Emamdadi et al. stated that the effects of magnetic and electromagnetic fields on HRP result from the interaction of these fields with the enzyme’s charged atoms [65]. These authors also noted that, in general, the effects of external fields can result from the changes in the spatial structure of enzymes [65]. A typical example of the latter case is lysozyme, whose spatial structure changes after exposure to either low-frequency (50 Hz) or microwave-frequency (900 MHz) electromagnetic fields [66].

The results obtained can be taken into consideration in designing biosensors and bioreactors, in which glycerol-containing heat transfer fluids are employed. In our experiments, we have demonstrated that triboelectric effects, initiated by glycerol flow, can lead to a generation of electromagnetic radiation. This radiation can have not only a standard topology but also a non-trivial one. HRP was reported to be sensitive to the radiation of non-trivial topology [20]. It can thus be used as a sensor for such radiation. We expect that our research report will influence the development of new biotechnological approaches and industrial processes by opening up possibilities for determining the influence of electromagnetic radiation on non-trivial topology on enzyme systems.

## 5. Conclusions

The use of AFM has allowed us to reveal a long-term effect of electromagnetic field, which was generated triboelectrically by the flow of glycerol, on the adsorption of HRP. Namely, increased content of the aggregated form of HRP has been observed on mica after a 40-min incubation of 0.1 µM buffered solution of the enzyme at a 2 cm distance from the ground-shielded coil with a stopped-flow of glycerol. We explain this fact by the impact of electromagnetic fields of non-trivial topology on the outer hydration shell of the enzyme globule. The enzymatic activity of HRP against its substrate ABTS remained unchanged, indicating no influence on the conformation of the enzyme’s active site. This is an important point since water is also known to be incorporated into active sites of enzymes [67]. Accordingly, in our experiments, only outer hydration shells have been affected. Considering the ubiquitous presence of electromagnetic fields in both industry and everyday life, we believe that the results of our study reported herein will help the researchers to better understand the role of these external factors in the functioning of enzyme systems.

## Data Availability

Data is contained within the article. The raw data supporting the conclusions of this article will be made available by the authors on request.
